# Incidence and prevalence of tuberculosis in systemic lupus erythematosus patients: A systematic review and meta-analysis

**DOI:** 10.3389/fimmu.2022.938406

**Published:** 2022-07-22

**Authors:** Qian Wu, Yang Liu, Wei Wang, Yu Zhang, Kui Liu, Song-Hua Chen, Bin Chen

**Affiliations:** ^1^ Department of Tuberculosis Control and Prevention, Zhejiang Provincial Center for Disease Control and Prevention, Hangzhou, China; ^2^ Department of Infectious Diseases Control and Prevention, Jiaxing Center for Disease Control and Prevention, Jiaxing, China

**Keywords:** systemic lupus erythematosus, tuberculosis, incidence, prevalence, glucocorticoids

## Abstract

**Background:**

Systemic lupus erythematosus (SLE) patients are particularly susceptible to infections, such as pulmonary tuberculosis (PTB) and extrapulmonary tuberculosis (EPTB). This meta-analysis aimed to determine the incidence and prevalence of tuberculosis (TB) in SLE patients.

**Methods:**

The Web of Science, PubMed, Cochrane Library, and Chinese National Knowledge Infrastructure databases were searched for articles of relevant studies published from the dates the databases were established until April 30, 2022. The *I^2^* statistic and *Q* test were used to evaluate heterogeneity among the analysed studies. Random-effects models were utilised and subgroup analyses were conducted for analysis of the study data.

**Results:**

A total of 35 studies with 46,327 SLE patients were eligible for analysis. The incidence and prevalence of TB among the SLE patients were 1.16 per 100 person-years (95% confidence interval (CI): 0.69-1.93) and 3.59% (95% CI: 2.57%-5.02%), respectively. The pooled prevalence of SLE-PTB and SLE-EPTB was 2.46% (95% CI: 1.73%-3.51%) and 1.42% (95% CI: 0.98%-2.06%), respectively. Subgroup analyses showed that the incidence of SLE-TB was higher in Africa and in countries with a high TB burden than in countries with a low TB burden. The prevalence of SLE-TB was elevated in Asia, in patients taking a mean daily dose of glucocorticoids ≥20 mg, in studies with small sample sizes (n <1000) and ended before 2001.

**Conclusions:**

The available evidence suggests that both the incidence and prevalence of TB in SLE patients are high. This study provides a more specific understanding of SLE-TB, which can help health policymakers in the development of preventive strategies for reducing the SLE-TB burden.

## 1 Introduction

Systemic lupus erythematosus (SLE) is a prototypical inflammatory autoimmune disease that involves multiple systems and is characterised by the accentuation of humoral immunity, excessive proliferation of B lymphocytes, and abnormal production of autoantibodies. The mechanisms underlying the aberrant immune responses associated with SLE remain elusive.

The findings of a previous meta-analysis suggest that adult patients with SLE have a 2-6 times higher risk of infection than that of the general population or healthy controls (1). Susceptibility to an extensive range of severe infections has become a major cause of morbidity and mortality in SLE patients, which imposes a serious public health burden (2, 3). The underlying reasons for this susceptibility to infections include impaired cellular immunity, defective phagocytic function, and use of glucocorticoids (GCs) and immunosuppressants (4).

Tuberculosis (TB) is a major infectious disease worldwide. In 2021, an estimated 9.87 million people developed TB and 1.50 million TB-related deaths were recorded (5). There is increasing evidence that SLE patients have an increased risk of developing pulmonary tuberculosis (PTB) or extrapulmonary tuberculosis (EPTB), particularly in countries with a high TB burden, including India, China, and South Africa (6–9). Despite the significance of this problem, no comprehensive systematic review and meta-analysis of published relevant studies has been conducted to ascertain the global incidence and prevalence of TB in SLE (SLE-TB) patients.

In recent years, studies have reported inconsistent results regarding the specific incidence and prevalence of SLE-TB (10–13). Therefore, a contemporary and comprehensive evaluation and update of the SLE-TB epidemiology data worldwide is necessary as it will provide a theoretical basis for TB screening and preventive treatment in SLE patients. In this meta-analysis, we systematically reviewed relevant literature to comprehensively quantify the incidence and prevalence of SLE-TB and offer insights into the potential factors responsible for heterogeneity in the reported estimates.

## 2 Methods

### 2.1 Search strategy

This meta-analysis was conducted according to the guidelines of the Preferred Reporting Items for Systematic Reviews and Meta-Analyses Statement. The Web of Science, PubMed, Cochrane Library, and Chinese National Knowledge Infrastructure databases were searched for articles of relevant studies published from the dates of the inception of the databases to April 30, 2022. A search strategy was designed for each electronic database. For example, the following search strategy was applied in the PubMed database: (“Tuberculosis” OR “TB” OR “pulmonary tuberculosis” OR “PTB” OR “extrapulmonary tuberculosis” OR “EPTB”) AND (“systemic lupus erythematosus” OR “lupus” OR “SLE”). The database search was conducted with no restriction on regions and languages.

### 2.2 Eligibility criteria

Studies that met the following criteria were included: (1) studies in which diagnostic verification was performed, SLE patients should be defined according to the American College of Rheumatology (ACR)-1982, ACR-1997 or International Classification of diseases, diagnosis of TB was made on the basis of clinical manifestations (fever, cough, sputum, dyspnea, hemoptysis, and weight loss), as well as radiological, bacteriological and histopathological evidence suggestive of TB; (2) studies that included relevant data on the incidence or prevalence of SLE-TB; (3) studies on adult patients with SLE; and (4) studies on patients diagnosed with TB after the onset of SLE. For studies with the same sample or duplicate populations, only the most recent study or the study with the largest sample size was included. Reviews, protocols, editorials, letters, and meeting abstracts were excluded.

### 2.3 Data extraction and quality assessment of included studies

After the primary literature search, all the articles were entered into the EndNote X7 software. First, the titles and abstracts of the articles were independently reviewed by two co-first authors and duplicate articles were excluded. Thereafter, the full texts of the remaining articles were reviewed and eligible articles were selected. Any disagreement during selection was resolved through a group discussion. The following characteristics of each eligible study were recorded: first author’s name, publication year, incidence of SLE-TB (number of SLE and SLE-TB patients, follow-up duration), prevalence of SLE-TB (or number of SLE and SLE-TB patients), the types of TB (PTB or EPTB), methods used for the diagnosis of SLE, mean daily dose of GCs, study period, country, region, TB burden, and study sites.

### 2.4 Statistical analysis

The *I^2^* statistic and *Q* test were used to evaluate heterogeneity among the includes studies. The pooled incidence and prevalence of SLE-TB (with 95% confidence intervals [CIs]) was defined as the number of SLE-TB patients per 100 person-years and per 100 persons (%), respectively. A random-effects model was used for analysis if *I^2^* was >50% and the *P* value for *Q* test was <0.10; a fixed-effects model was used if otherwise. To identify the possible source of heterogeneity among the studies, subgroup analysis was conducted according to region, TB burden, diagnostic criteria for SLE, sample size, and mean daily dose of GCs. Publication bias was assessed using the Egger’s and Begg’s tests. Sensitivity analysis was conducted by iteratively removing one study from the meta-analysis. All statistical analyses were conducted using R software (version 3.6.1; http://www.R-project.org) with the “meta” package. Statistical significance was set at *P* < 0.05.

## 3 Results

### 3.1 Search results

**Figure 1:**
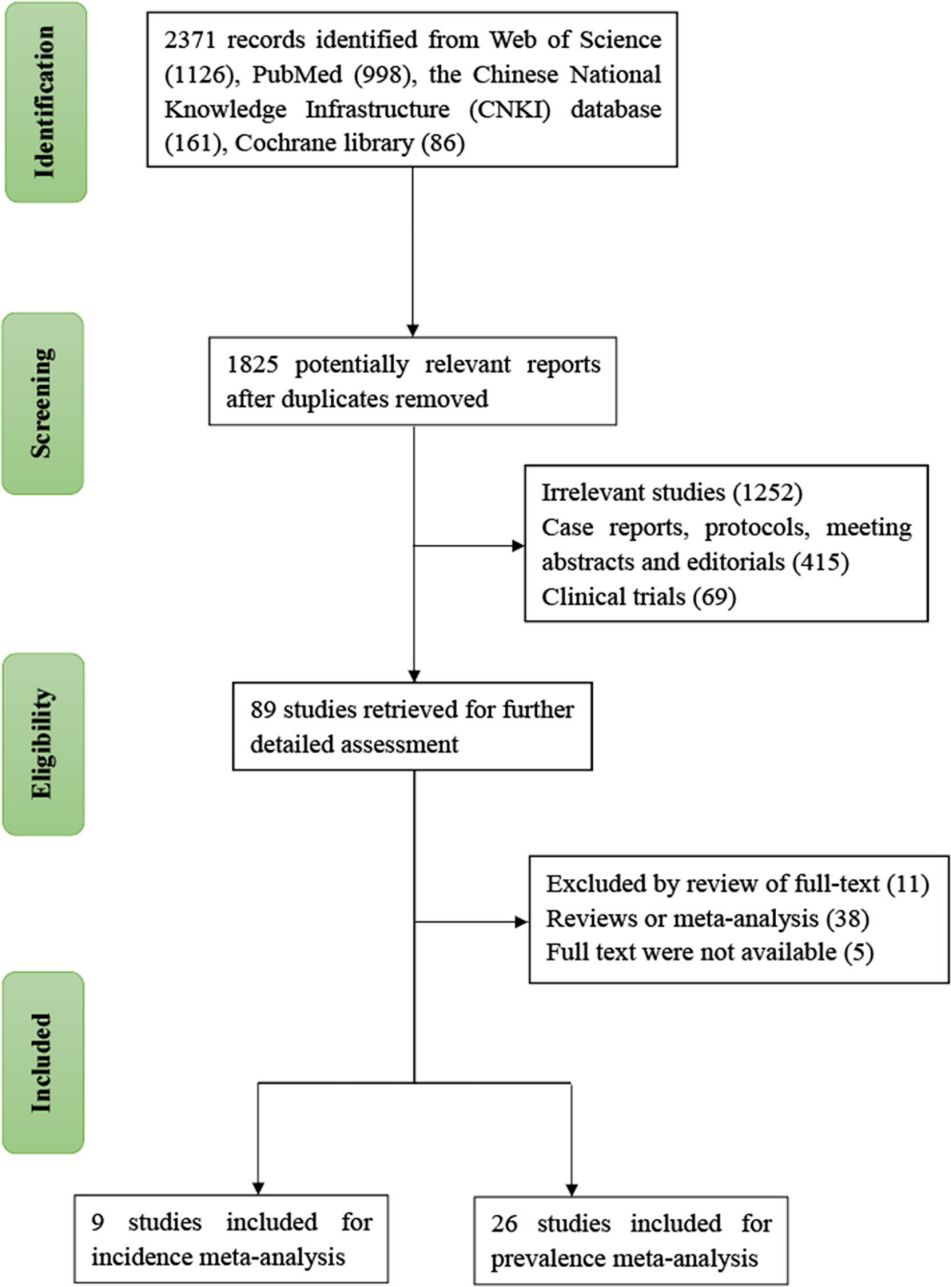
Flow chart of study selection.

After a comprehensive literature search, 2371 articles from the Web of Science (1,126), PubMed (998), Cochrane library (86), and Chinese National Knowledge Infrastructure (161) databases were initially retrieved. Of these, 1825 relevant studies were identified based on the abovementioned eligibility and exclusion criteria. After manually reviewing the full texts, 1736 studies were excluded. Finally, 35 eligible studies, including nine cohort and 26 cross-sectional studies with a total of 46,327 SLE patients were included in the meta-analysis (6–40) **(** **Figure 1** **)**. Of the included studies, 25 were published in English, eight in Chinese, and two in Spanish. All included studies were published between 1982 and 2021. The sample sizes ranged from 141 to 10469 participants. The baseline and general characteristics of the included studies are shown in **Tables 1**, **2**.

**Table 1:** Summary of the studies that reported the TB incidence among SLE patients.

Study	Country	Region	SLE-TB	Sample size	PTB	EPTB	Mean age	Period	TB-burden	SLE criteria
Victorio-Navarra ST 1996	Philippines	Asia	54	390	42	NR	32	1985-1995	High	ACR-1982
Yun JE 2002	Korea	Asia	15	283	12	6	36.1	1979-2000	Not high	ACR-1982
Tam LS 2002	Hong Kong, China	Asia	57	526	19	38	36	1984-2001	High	ACR-1997
Mok MY 2003	Hong Kong, China	Asia	91	652	55	36	31.6	1987-2001	High	ACR-1982
Vadillo 2003	Spain	Europe	3	158	2	1	53	1991-2000	Not high	NR
Erdozain JG 2006	Spain	Europe	3	221	3	0	33	1994-2003	Not high	ACR-1997
Hodkinson B 2009	South Africa	Africa	97	568	81	30	34.9	1989-2006	High	ACR-1997
Muhammed H 2021	India	Asia	48	1335	27	21	27.6	NR	High	ACR-1997
Gupta L 2021	India	Asia	8	131	4	4	32.4	2015-2020	High	ACR-1997

TB, tuberculosis; PTB, pulmonary tuberculosis; NR, not reported; EPTB, extrapulmonary tuberculosis; ACR, American College of Rheumatology.

**Table 2:** Summary of the studies that reported the TB prevalence among SLE patients.

Study	Country	Region	SLE-TB	Sample size	PTB	EPTB	Mean age	Period	TB-burden	SLE criteria
Feng PH 1982	Singapore	Asia	16	311	13	3	29	1963-1979	Not high	ACR-1982
Shyam C 1996	India	Asia	41	309	32	9	28.8	1989-1994	High	ACR-1982
Balakrishnan C 1998	India	Asia	17	146	14	NR	NR	NR	High	NR
Kim HY 1999	Korea	Asia	22	256	22	0	34	1989-1997	Not high	ACR-1982
Sayarlioglu M 2004	Turkey	Asia	20	556	11	9	34	1978-2001	Not high	NR
Pan JP 2005	China	Asia	18	426	12	6	30.5	1991-2004	High	ACR-1982
Lei XM 2005	China	Asia	42	915	32	18	28	1995-2004	High	ACR-1982
Liu G 2006	China	Asia	32	1678	28	9	34.8	2002-2003	High	ACR-1982
Zhang R 2007	China	Asia	93	2682	73	48	29.4	1997-2006	High	ACR-1982
Zhang L 2008	China	Asia	42	452	11	31	34.9	1997-2006	High	ACR-1997
Hou CL 2008	Taiwan,China	Asia	21	3179	10	11	45	1985-2004	High	ACR-1982
Chu AD 2009	USA	North America	6	187	5	4	45.2	2005	Not high	ACR-1997
Wang J 2009	China	Asia	41	1245	NR	NR	40.25	1990-2001	High	ACR-1982
González León R 2010	Spain	Europe	13	789	9	8	36	1980-2009	Not high	NR
Pasoto SG 2010	Brazil	South America	20	1283	20	0	41.9	2001-2009	High	ACR-1997
Ma L 2014	China	Asia	8	203	4	4	32	2007-2011	High	ACR-1997
Yang Y 2017	Singapore	Asia	17	841	14	3	53.9	2004-2011	Not high	NR
Zhan ZP 2017	China	Asia	42	782	28	25	31.3	NR	High	ACR-1997
Torres-González P 2018	Mexico	North America	72	5365	38	48	35	1990-2014	Not high	ACR-1982
Lao M 2019	China	Asia	59	1108	41	18	34.4	2007-2017	High	ACR-1997
Song WY2019	China	Asia	7	141	5	4	35.14	2016-2019	High	NR
Hamijoyo L 2019	Indonesia	Asia	93	813	NR	NR	27.7	2016-2017	High	ACR-1997
Ahmmed MF 2019	Bangladesh	Asia	23	230	15	8	NR	2015-2016	High	NR
González–Naranjo LA 2021	USA	North America	67	4738	54	31	34	2007-2017	Not high	ACR-1997
Xiao X 2021	China	Asia	158	10469	103	NR	37	1983-2019	High	NR
Liu X 2021	China	Asia	41	2959	NR	20	42	2014-2016	High	NR

TB, tuberculosis; PTB, pulmonary tuberculosis; NR, not reported; EPTB, extrapulmonary tuberculosis; ACR, American College of Rheumatology.

### 3.2 Incidence of SLE-TB

**Figure 2:**
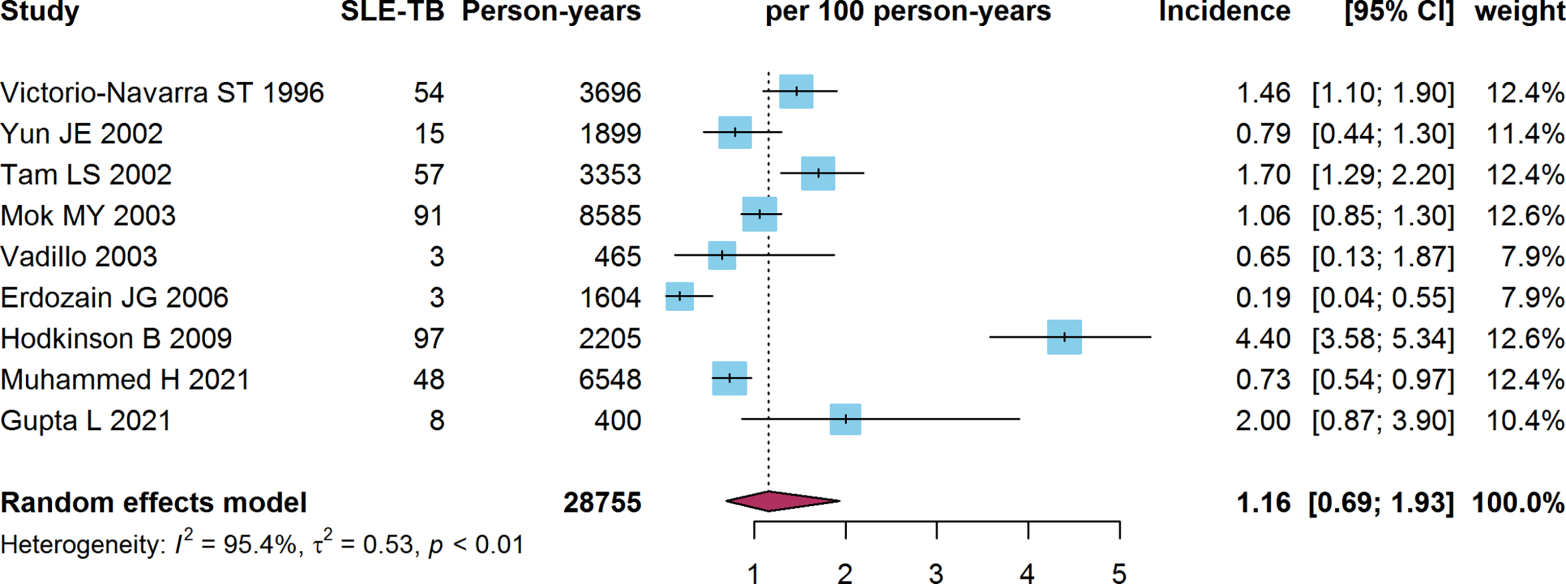
Forest plot of the SLE-TB incidence. SLE, systemic lupus erythematosus; TB, tuberculosis.

The incidence of TB in SLE patients was reported in nine studies, which included 4,264 SLE patients with 28,755 person-years of follow-up. The pooled estimate of the incidence of SLE-TB in these studies was 1.16 (95% CI: 0.69-1.93) per 100 person-years **(** **Figure 2** **)**. Statistically significant heterogeneity was observed between the studies (I^2 =^ 95.4%, τ^2 =^ 0.53, *P*<0.01).

#### 3.2.1 Subgroup analyses of the SLE-TB incidence

The subgroup meta-analysis stratified according to TB burden revealed that incidence of SLE-TB in countries with a high TB burden (1.60 per 100 person-years, 95% CI: 0.89-2.86) was higher than that in countries with a low TB burden (0.50 per 100 person-years, 95% CI: 0.22-1.17) (*P*=0.03). Only one of the studies was conducted in Africa. The pooled prevalence of SLE-TB in Africa was higher (4.40 per 100 person-years, 95% CI: 3.62-5.34) than that in Asia (1.18 per 100 person-years, 95% CI: 0.88-1.59) and Europe (0.35 per 100 person-years, 95% CI: 0.10-1.17) (*P*<0.01). However, the incidence of SLE-TB according to the 1982 criteria of the American College of Rheumatology (ACR-1982) or ACR-1997 did not differ between subgroups (*P*=0.79). Besides, no significant difference in the SLE-PTB incidence was observed when stratified according to study period of these studies (*P*=0.91) (**Table 3**)

**Table 3:** Subgroup analysis of TB incidence and prevalence among SLE patients.

Subgroups	N	Effect size [95% CI]	*I^2^*	*P-value* of between-subgroup heterogeneity
Incidence (per 100 person-years)				
Region				<0.01
Asia	6	1.18(0.88,1.59)	81.4%	
Europe	2	0.35(0.10,1.17)	56.7%	
Africa	1	4.40(3.62,5.34)	–	
TB burden				0.03
High	6	1.60(0.89,2.86)	96.6%	
Not high	3	0.50(0.22,1.17)	61.6%	
Diagnostic criteria of SLE				0.79
ACR-1982	3	1.12(0.84,1.51)	66.0%	
ACR-1997	5	1.27(0.54,2.98)	96.9%	
Study period				0.91
Before 2001	3	1.03(0.60,1.75)	65.7%	
2001-2010	4	1.28(0.55,3.02)	97.5%	
2011-2020	2	1.15(0.43,3.06)	85.8%	
Prevalence				
Region				<0.01
Asia	21	4.27%(2.97%,6.14%)	96.6%	
Europe	1	1.65%(0.96%,2.82%)	–	
North America	3	1.50%(1.14%,1.98%)	54%	
South America	1	1.56%(1.01%,2.41%)	–	
TB burden				0.22
High	18	4.05%(2.70%,6.07%)	97.1%	
Not high	8	2.71%(1.65%,4.46%)	92.6%	
Sample size				<0.01
<1000	16	5.61%(4.17%,7.55%)	89.7%	
≥1000	10	1.87%(1.32%,2.65%)	94.5%	
Mean daily dose of GCs				0.04
<20	4	1.48%(1.27%,1.72%)	34.7%	
≥20	6	3.06%(1.54%,6.06%)	95.1%	
Diagnostic criteria of SLE				0.63
ACR-1982	10	3.42%(2.04%,5.73%)	96.4%	
ACR-1997	8	4.18%(2.25%,7.76%)	96.9%	
Study period				<0.01
Before 2001	4	9.31%(6.25%,13.86%)	75.4%	
2001-2010	11	2.81%(1.88%,4.19%)	92.8%	
2011-2020	11	3.28%(1.88%,5.72%)	97.8%	

TB, tuberculosis; SLE, systemic lupus erythematosus; N, number; CI, confidence intervals; GCs, glucocorticoids; ACR, American College of Rheumatology.

#### 3.2.2 Sensitivity analysis and publication bias

Sensitivity analysis revealed no significant changes in the overall estimates of the incidence of SLE-TB after omitting any study **(** **Figure S1** **)**. The results of the Egger’s (t= -1.22, *P*=0.260) and Begg’s (z= -0.83, *P*=0.404) tests indicated no evidence of publication bias in the pooled incidence of SLE-TB **(** **Figure S2** **)**.

### 3.3 Prevalence of SLE-TB

**Figure 3:**
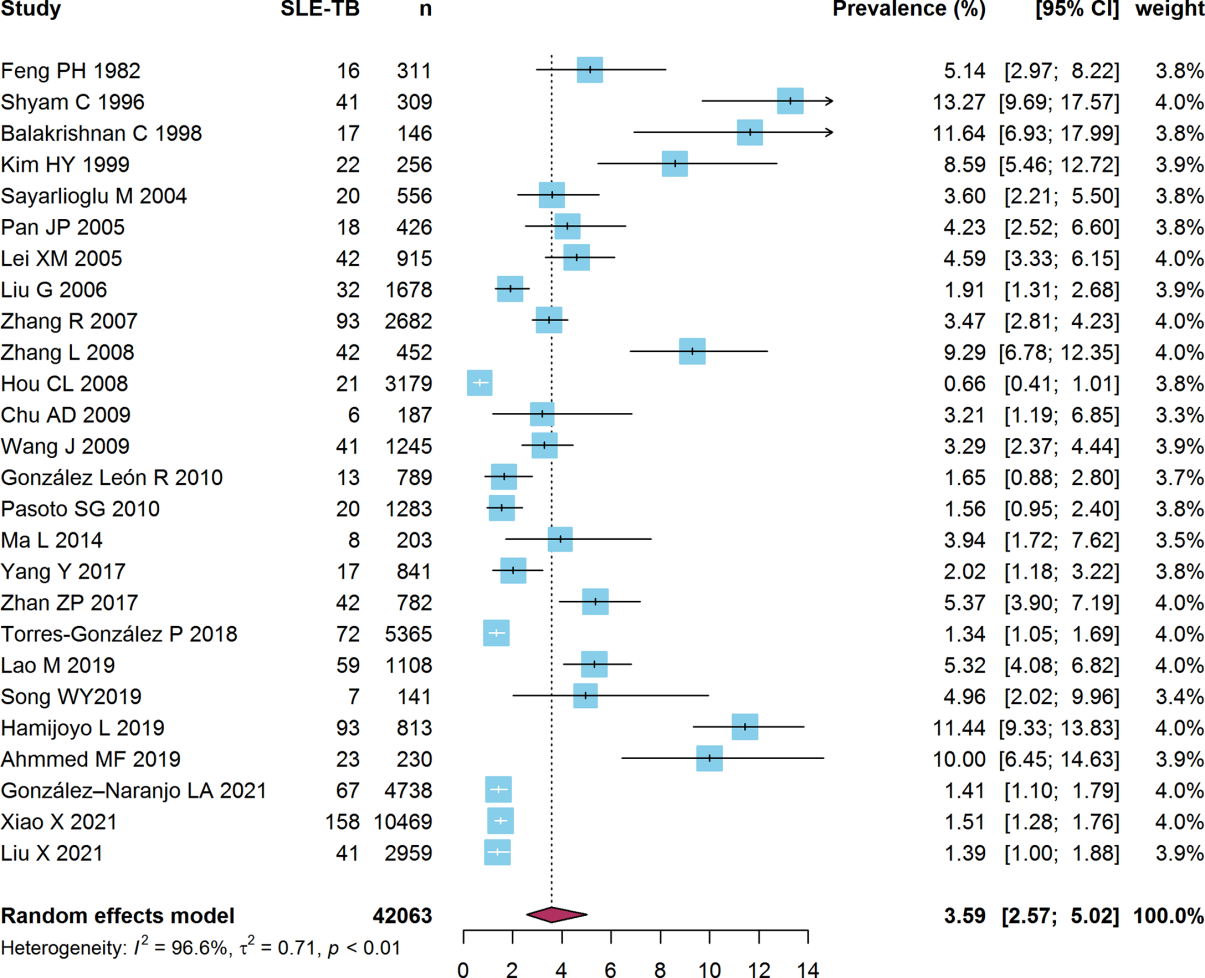
Forest plot of the SLE-TB prevalence. SLE, systemic lupus erythematosus; TB, tuberculosis.

The prevalence of TB in SLE patients was reported in 26 studies, which included 42,063 SLE patients. The pooled estimate of the prevalence of SLE-TB in the studies was 3.59% (95% CI: 2.57%-5.02%) **(** **Figure 3** **)**. Heterogeneity among these studies was statistically significant (I^2 =^ 96.6%, τ^2 =^ 0.71, *P*<0.01). The prevalence of SLE-PTB was reported in 23 studies, which included 37,046 SLE patients. The pooled prevalence of SLE-PTB in the studies was 2.46% (95% CI: 1.73%-3.51%). There was high heterogeneity among these studies as well (I^2 =^ 94.6%, τ^2 =^ 0.68, *P*<0.01) **(** **Figure S3** **)**. The prevalence of SLE-EPTB was reported in 20 studies with 27,851 SLE patients. The pooled prevalence of SLE-EPTB in the studies was 1.42% (95% CI: 0.98%-2.06%) and high heterogeneity was noted among the studies (I^2 =^ 90.3%, τ^2 =^ 0.60, *P*<0.01) **(** **Figure S4** **)**.

#### 3.3.1 Subgroup analyses of the prevalence of SLE-TB

Only one study each was conducted in Europe and South America. The pooled prevalence of SLE-TB in Asia was higher (4.27%, 95% CI: 2.97%-6.14%) than that in Europe (1.65%, 95% CI: 0.96%-2.82%), South America (1.56%, 95% CI: 1.01%-2.41%), and North America (1.50%, 95% CI: 1.14%-1.98%) (*P*<0.01). The subgroup meta-analysis stratified according to sample size showed that the prevalence of SLE-TB in studies with a small sample size (n <1000) (5.61%, 95% CI: 4.17%-7.55%) was three times higher than that in studies with a large sample size (n ≥1000) (1.87%, 95% CI: 1.32%-2.65%) (*P*<0.01). The pooled prevalence of SLE-TB among patients taking a mean daily dose of GCs ≥20 mg was higher (3.06%, 95% CI: 1.54%-6.06%) than that in patients taking a mean daily dose of GCs <20 mg (1.48%, 95% CI: 1.27%-1.72%) (*P* = 0.04). In terms of study period of these studies, the SLE-TB prevalence in studies ended before 2001 (9.31%, 95% CI: 6.25%-13.86%) was significantly higher than that in studies ended between 2001-2010 and 2011-2020 (*P*<0.01). However, no significant difference in the prevalence of SLE-PTB was observed when stratified according to TB burden (*P*=0.22) and diagnostic criteria of SLE (*P*=0.63) (**Table 3**).

#### 3.3.2 Sensitivity analysis and publication bias

Sensitivity analysis revealed no significant changes in the overall estimates of the prevalence of SLE-TB after omitting any study **(** **Figure S5** **)**. The results of the Egger’s (t = 0.51, *P* = 0.615) and Begg’s (z = -0.20, *P* = 0.843) tests indicated no evidence of publication bias in the pooled prevalence of SLE-TB **(** **Figure S6** **)**.

## 4 Discussion

Previous studies conducted in different countries have shown that SLE patients had a 5-60-fold higher risk of TB than that of patients without (15, 17, 18, 33, 39). In this systematic review, we analysed data from 35 studies, which included 46,327 SLE patients from 13 countries across five continents. The results of the meta-analysis indicated that the incidence and prevalence of TB among SLE patients was 1.16 per 100 person-years (95% CI: 0.69-1.93) and 3.59% (95% CI: 2.57%-5.02%), respectively. Numerous studies have presented convincing biological evidence supporting a causal relationship between compromised immune defence systems and susceptibility to TB (41, 42). SLE patients exhibit atypical clinical manifestations of TB, including fever, arthralgia, pleurisy, peritonitis, and headache. Moreover, a retrospective study revealed that the radiological manifestations and laboratory results of TB in SLE patients are relatively atypical (35). In this regard, timely diagnosis and treatment of TB in SLE patients may be difficult to determine, reinforcing the need for disease surveillance in this population.

Several studies have demonstrated that SLE patients exhibit relatively frequent extrapulmonary involvement, indicating the reactivation of past infections rather than the occurrence of new primary infections (17, 27, 36). Conversely, the results of the present study revealed a relatively higher prevalence of SLE-PTB (2.46%, 95% CI: 1.73%-3.51%) than that of SLE-EPTB (1.42%, 95% CI: 0.98%-2.06%). Our findings conform with those of a retrospective case-control study in which PTB accounted for 46.3% of all cases, whereas EPTB and disseminated TB accounted for 16.4% and 37.3%, respectively (13).

The subgroup analyses in the present study revealed significant differences between the incidence and prevalence of SLE-EPTB in the five aforementioned continents. The variations among the studies conducted in these continents may be attributable to differences in the prevention strategies employed in the countries and the ethnic and sociodemographic statuses of the study participants (43, 44). Another possible explanation for the higher prevalence of SLE-TB in Asia than in Europe, South America, and North America could be the relatively larger proportion of high TB burden countries in Asia, such as India and China.

We found that the pooled incidence of TB was significantly higher in countries with a high TB burden. However, it should be noted that more than three-quarters of the studies included in this meta-analysis were conducted in endemic regions. Thus, large-scale, prospective, studies performed in several countries are required to obtain more reliable estimates of the global incidence of the SLE-TB.

A high cumulative and/or mean daily dose of GCs has been implicated as a predisposing factor for the development of TB in SLE patients (17, 21, 45). For example, a case-control study indicated that patients taking GCs have an approximately five-fold increased risk of developing TB compared to those who are not taking GCs (46). Consistent with these findings, we found that the pooled prevalence of SLE-TB in patients with a mean daily dose of GCs ≥20 mg was significantly higher than that in patients with a mean daily dose of GCs <20 mg. The exact mechanism of the effect of GC therapy in SLE patients has not been fully elucidated. However, it may involve the decline of cellular immune function through the inhibition of lymphocytes, monocyte macrophages, neutrophils, and eosinophils. Thus, administration of a minimum dose of GCs is recommended for better control of TB.

This systematic review had several limitations. First, the heterogeneity among the included studies was significant. This might have been caused by differences in populations and geographic regions. Therefore, the pooled incidence and prevalence of the diseases indicated in the results should be interpreted with caution. Second, previous studies have shown that the Systemic Lupus Erythematosus Disease Activity Index, use of immunosuppressants, duration of treatment, and presence of nephritis are risk factors for developing TB (9, 17, 44). Unfortunately, we were unable to retrieve sufficient information regarding these variables from most of the included studies. Third, there may be selection bias in the results owing to the retrospective nature of some of the included cohort studies. Thus, future, well-designed, prospective studies conducted using standardised methods of data collection and reporting are required to clarify the incidence of TB in SLE patients.

These limitations notwithstanding, this meta-analysis comprehensively summarises the current evidence regarding the global incidence and prevalence of TB in SLE patients, and provides a reference that can be used in the prevention and management of TB in SLE patients. Efforts to improve the understanding of SLE-TB and develop preventive strategies against it are required in the future.

## 5 Conclusion

The available evidence indicates that the incidence and prevalence of TB among SLE patients are significantly higher than those in the general population. More attention should be paid to strengthening strategies aimed at preventing TB and developing better treatments for SLE, especially in countries with a high TB burden and in patients taking a mean daily dose of GCs ≥20 mg.

## Data availability statement

The original contributions presented in the study are included in the article/**Supplementary Material**. Further inquiries can be directed to the corresponding author.

## Author contributions

QW, YL and BC conceptualized the meta-analysis, QW and YL conducted the research and performed the statistical analysis. QW drafted the initial manuscript. All authors contributed to the article and approved the submitted version.

## Funding

This study was supported by the National-Zhejiang Health commission Major S&T Project (Grant No. WKJ-ZJ-2118), Zhejiang Provincial Medical and Health Project (2021KY618 and 2020KY520).

## Conflict of interest

The authors declare that the research was conducted in the absence of any commercial or financial relationships that could be construed as a potential conflict of interest.

## Publisher’s note

All claims expressed in this article are solely those of the authors and do not necessarily represent those of their affiliated organizations, or those of the publisher, the editors and the reviewers. Any product that may be evaluated in this article, or claim that may be made by its manufacturer, is not guaranteed or endorsed by the publisher.
