# Supplementary material for: Incidence and prevalence of tuberculosis in systemic lupus erythematosus patients: A systematic review and meta-analysis
Source: Front Immunol. 2022 Jul 22;13:938406. doi: 10.3389/fimmu.2022.938406 (PMC9355093; doi:10.3389/fimmu.2022.938406)
Supplement: Supplementary file 1 [file DataSheet_1.doc]

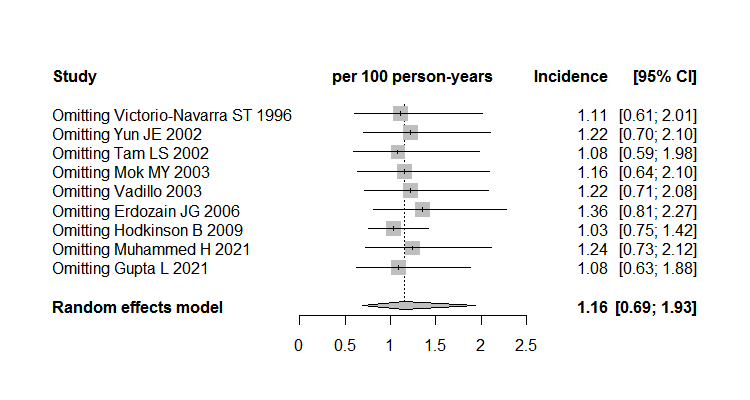


**Figure S1.** Sensitivity analyses for studies reporting the incidence of SLE-TB.


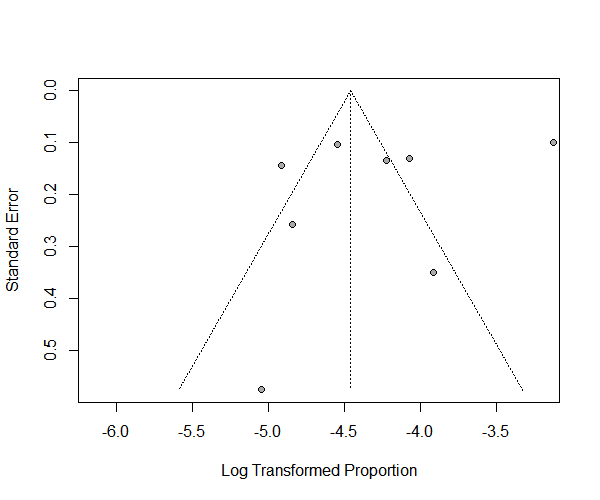


**Figure S2.** Funnel plot for studies reporting the incidence of SLE-TB.


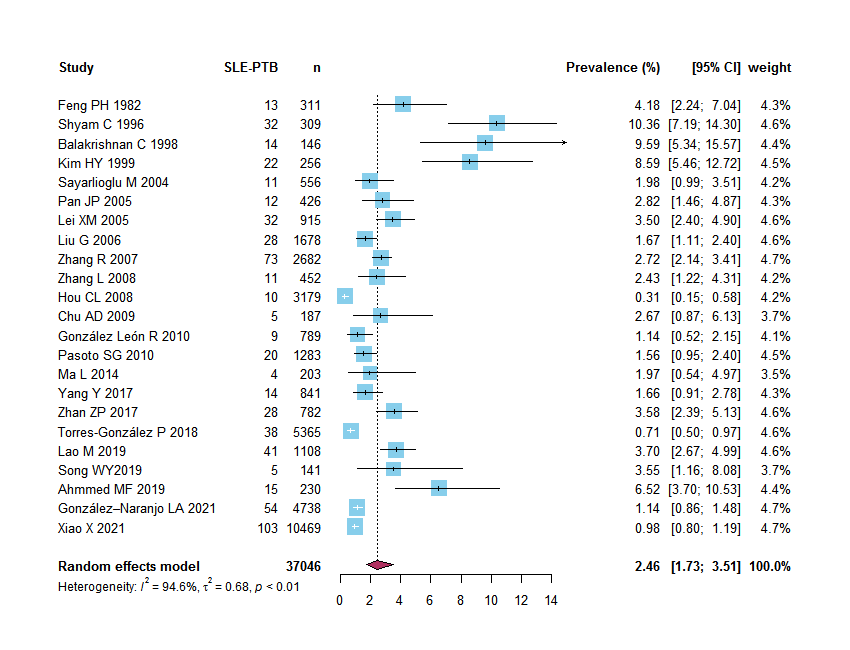


**Figure S3.** Forest plot showing the prevalence of SLE-PTB.


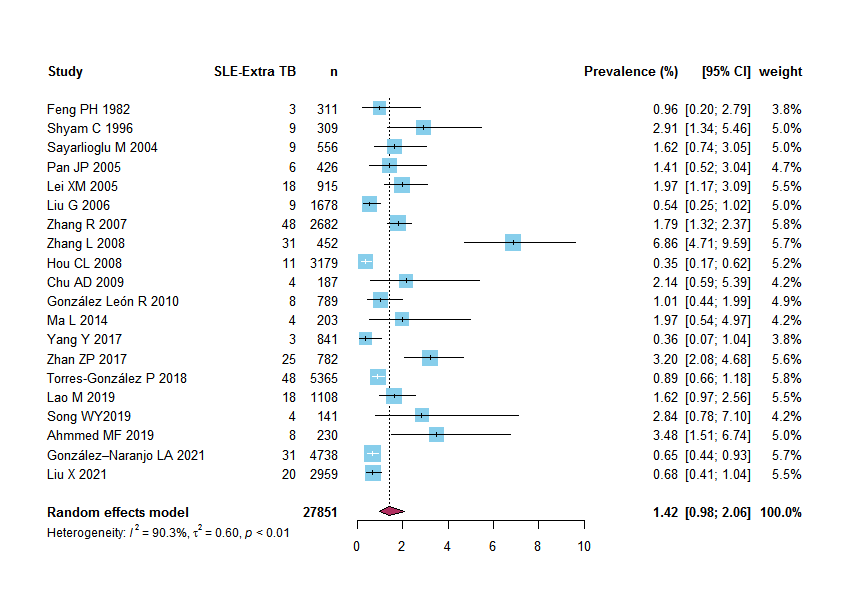


**Figure S4.** Forest plot showing the prevalence of SLE-EPTB.


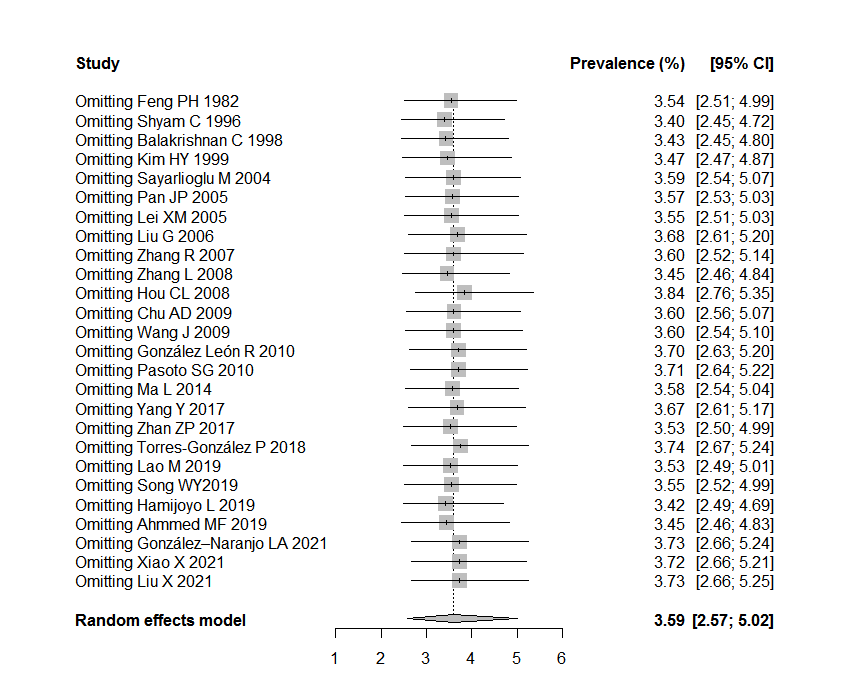


**Figure S5.** Sensitivity analyses for studies reporting the prevalence of SLE-TB.


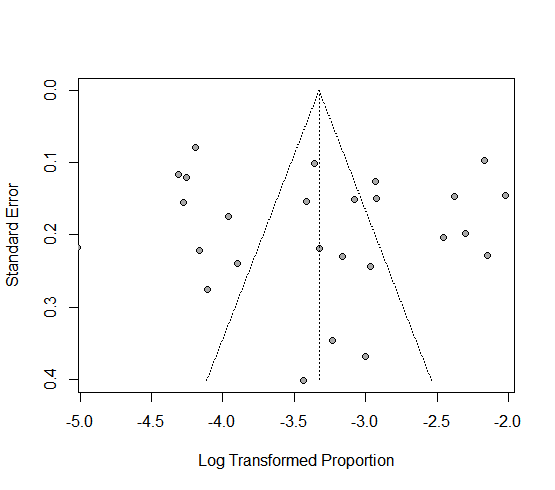


**Figure S6.** Funnel plot for studies reporting the prevalence of SLE-TB.
